# Ciglitazone—a human PPARγ agonist—disrupts dorsoventral patterning in zebrafish

**DOI:** 10.7717/peerj.8054

**Published:** 2019-11-13

**Authors:** Vanessa Cheng, Subham Dasgupta, Aalekhya Reddam, David C. Volz

**Affiliations:** Department of Environmental Sciences, University of California, Riverside, CA, USA

**Keywords:** PPARγ, Ciglitazone, Dorsoventral patterning, Zebrafish, Embryo

## Abstract

Peroxisome proliferator-activated receptor γ (PPARγ) is a ligand-activated transcription factor that regulates lipid/glucose homeostasis and adipocyte differentiation. While the role of PPARγ in adipogenesis and diabetes has been extensively studied, little is known about PPARγ function during early embryonic development. Within zebrafish, maternally-loaded pparγ transcripts are present within the first 6 h post-fertilization (hpf), and de novo transcription of zygotic pparγ commences at ~48 hpf. Since maternal pparγ transcripts are elevated during a critical window of cell fate specification, the objective of this study was to test the hypothesis that PPARγ regulates gastrulation and dorsoventral patterning during zebrafish embryogenesis. To accomplish this objective, we relied on (1) ciglitazone as a potent PPARγ agonist and (2) a splice-blocking, pparγ-specific morpholino to knockdown pparγ. We found that initiation of ciglitazone—a potent human PPARγ agonist—exposure by 4 hpf resulted in concentration-dependent effects on dorsoventral patterning in the absence of epiboly defects during gastrulation, leading to ventralized embryos by 24 hpf. Interestingly, ciglitazone-induced ventralization was reversed by co-exposure with dorsomorphin, a bone morphogenetic protein signaling inhibitor that induces strong dorsalization within zebrafish embryos. Moreover, mRNA-sequencing revealed that lipid- and cholesterol-related processes were affected by exposure to ciglitazone. However, pparγ knockdown did not block ciglitazone-induced ventralization, suggesting that PPARγ is not required for dorsoventral patterning nor involved in ciglitazone-induced toxicity within zebrafish embryos. Our findings point to a novel, PPARγ-independent mechanism of action and phenotype following ciglitazone exposure during early embryonic development.

## Introduction

Peroxisome proliferator-activated receptor gamma (PPARγ) is a nuclear receptor that, upon activation by endogenous (e.g., fatty acids, prostaglandins) or exogenous (e.g., thiazolidinediones) ligands, heterodimerizes with retinoid X receptor and binds to PPAR response elements in order to regulate transcription of genes such as adipocyte fatty acid binding protein (A-FABP/aP2) and cytochrome P450 4B1 (CYP4B1) (Issemann & Green, 1990; Kliewer et al., 1997). PPARγ plays a central role in lipid/glucose homeostasis, adipocyte differentiation, proliferation, and immune response (Chawla et al., 1994; Tontonoz, Hu & Spiegelman, 1994; Ricote et al., 1998; Martin et al., 1998). As a mediator of adipogenesis, PPARγ plays a role in the progression of pathological diseases such as obesity, diabetes, atherosclerosis, cancer, and chronic inflammation (Vidal-Puig et al., 1996; Tontonoz et al., 1997; Gilroy et al., 1999). As such, PPARγ is a promising target for small molecule drugs. For example, PPARγ agonists (e.g., rosiglitazone and pioglitazone) have been used for nearly 20 years for treatment of type II diabetes mellitus (Lehmann et al., 1995; Martens et al., 2002).

While the expression and function of PPARγ has mainly been studied within adult adipose, muscle, heart, pancreatic, and liver tissue, several studies have demonstrated that PPARγ also plays a role in normal development and is expressed within human primary trophoblast cells and placental tissue (Storvik et al., 2014; El Dairi et al., 2018). Based on PPARγ knockout mice, PPARγ is essential for trophoblast differentiation and placental vascularization, and embryos lacking either of these processes leads to myocardial thinning and, ultimately, prenatal death (Barak et al., 1999). Based on ruminant and porcine studies, PPARγ also plays a role in conceptus elongation, the process by which the trophoblast of the spherical blastocyst elongates, differentiates, and secretes products that change the physiology of the endometrium for implantation and placental development (Brooks, Burns & Spencer, 2015; Ribeiro et al., 2016; Blitek & Szymanska, 2019). Within Western clawed frog (*Xenopus tropicalis*) embryos, knockdown of PPARγ by morpholino (MO) injection results in defects of eye development as well as disruption of lipid and glucose homeostasis (Zhu et al., 2018).

Dorsoventral patterning—a highly-conserved process that governs how dorsal and ventral structures are determined within a vertebrate and invertebrate embryo—is controlled by a complex array of maternal and zygotic factors and signaling pathways, including retinoic acid, Wnt, fibroblast growth factor, Sonic hedgehog, and bone morphogenetic protein (BMP) signaling (Chazaud et al., 1996; Furthauer, Thisse & Thisse, 1997; Furthauer et al., 2004; Chiang et al., 1996; Kishimoto et al., 1997). A gradient of BMP agonists (e.g., Bmp2b/7) and BMP antagonists (e.g., chordin) organize the development and differentiation of cells into ventral and dorsal structures, respectively (Dick et al., 2000). As an embryo progresses through development, strict regulation of these various factors is required for proper dorsoventral patterning. Indeed, this process is sensitive to environmental chemicals that disrupt signaling pathways regulating dorsoventral patterning, resulting in dorsalized or ventralized embryos (Dasgupta et al., 2017).

Within the first 24 h post-fertilization (hpf), zebrafish embryos rapidly progress through cleavage, blastula, gastrula, and segmentation (Kimmel et al., 1995), resulting in a properly formed embryo with dorsal and ventral structures by 24 hpf. Within zebrafish, maternally-loaded PPARγ transcripts are only present within the first 6 hpf, and de novo transcription of zygotic PPARγ does not commence until ~48 hpf (White et al., 2017). Within mice and in vitro studies, PPARγ is known to interact with BMP signaling during differentiation of mesenchymal cells into different osteogenic cell fates (Nishii et al., 2009; Shen et al., 2010; Stechschulte et al., 2016; Chung et al., 2016; Wang et al., 2018). Therefore, since maternal PPARγ transcripts are elevated during a critical window of cell fate specification, the objective of this study was to test the hypothesis that PPARγ regulates gastrulation and dorsoventral patterning during zebrafish embryogenesis.

## Materials and Methods

### Animals

Adult wildtype (strain 5D) zebrafish were maintained and bred on a recirculating system using previously described procedures (Mitchell et al., 2018). All adult breeders were handled and treated in accordance with Institutional Animal Care and Use Committee-approved animal use protocols (#20150035 and #20180063) at the University of California, Riverside.

### Chemicals

Ciglitazone (>99.4% purity) was purchased from Tocris Bioscience (Bristol, UK), and dorsomorphin (DMP) (99.7% purity) was purchased from Millipore Sigma (St. Louis, MO, USA). For both chemicals, stock solutions were prepared in high performance liquid chromatography-grade dimethyl sulfoxide (DMSO) and stored in two mL amber glass vials with polytetrafluoroethylene-lined caps. Working solutions were prepared by spiking stock solutions into particulate-free water from our recirculating system (pH and conductivity of ~7.2 and ~950 μS, respectively) immediately prior to each experiment, resulting in 0.2% DMSO within all vehicle control and treatment groups. Propylene glycol (>99.5% purity) and Oil Red O (ORO) (>75% dye content) were purchased from Fisher Scientific (Hampton, NH, USA) and Sigma–Aldrich (St. Louis, MO, USA), respectively.

### Bioinformatics

Zebrafish-specific pparγ, pparαa, pparαb, pparδa, and pparδb transcript abundance (transcripts per million, or TPM) across developmental stages were obtained from White et al. (2017) (provided within White et al., 2017 as Supplementary File 3, RNA-seq TPM; .tsv file), and stages were converted into hpf per Kimmel et al. (1995). Five replicate TPM values per developmental stage were used to calculate the mean TPM ± standard deviation at each developmental stage. PPARα, PPARδ, and PPARγ amino acid sequences for *Homo sapiens* (NP_005027.2; NP_006229.1; NP_056953.2), *Mus musculus* (NP_035274.2; NP_035275.1; NP_035276.2), *Rattus norvegicus* (NP_037328.1; NP_037273.2; NP_001138838.1), and *Danio rerio* (NP_001154805.1 (αa); NP_001096037.1 (αb); XP_699900.6 (δa); NP_571543.1 (δb); NP_571542.1) were obtained from the National Center for Biotechnology Information (www.ncbi.nlm.nih.gov). Sequences were aligned using the Multiple Sequence Alignment Tool within Clustal Omega (https://www.ebi.ac.uk/Tools/msa/clustalo/), and the aligned file was used to generate a cladogram within Clustal Omega. Pairwise sequence alignments were also performed to obtain percent amino acid similarity using EMBOSS Matcher (https://www.ebi.ac.uk/Tools/psa/emboss_matcher/). The following default options were used for all pairwise alignments: Matrix = BLOSUM62; Gap Open = 1; Gap Extend = 4; and Alternatives = 1.

### Embryo exposures and phenotyping

Embryos were sorted and exposed to either vehicle (0.2% DMSO) or ciglitazone (9.375, 12.5, 15, or 20 μM) from 4 to 24 hpf in glass petri dishes (20 embryos per replicate; three replicates per treatment). Ciglitazone concentrations were selected based on the maximum tolerated concentration (based on survival as an endpoint) in zebrafish embryos following a 4–24 hpf exposure. At 24 hpf, embryos were imaged under transmitted light at 2× magnification using a Leica MZ10 F stereomicroscope equipped with a DMC2900 camera and assessed for survival and dorsoventral patterning abnormalities (ventralization, dorsalization, or delayed development). Following previously described protocols (Dasgupta et al., 2017), ventralized embryos were defined as embryos with a swollen yolk sac extension; dorsalized embryos were defined as embryos with a tail deformity; and delayed embryos were defined as embryos that phenocopied embryos at a developmental stage prior to 24 hpf.

### Morpholino injections

Morpholino antisense oligos were synthesized and obtained from Gene Tools, Inc. (Philomath, OR, USA). A fluorescein-tagged splice-blocking MO was designed to target the first exon-intron boundary (E1I1) of zebrafish pparγ-specific pre-mRNA (NCBI Gene ID: 557037), leading to insertion of intron 1 within pparγ mRNA (pparγ-MO sequence: 5′-TCAGCTCCTCTCTGACACTTACCAG-3′). We did not rely on a pparγ-specific translational MO due to the lack of a commercially available PPARγ-specific antibody that cross reacts with zebrafish PPARγ and, as such, inability to confirm knockdown of PPARγ protein. Gene Tools’ standard fluorescein-tagged negative control MO (nc-MO)—a MO that targets a human β-globin intron mutation—was used in order to account for potential non-target MO toxicity, and a zebrafish-specific, fluorescein-tagged chordin MO (chd-MO sequence: 5′-ATCCACAGCAGCCCCTCCATCATCC-3′) was used as a positive control for disruption of dorsoventral patterning (ventralization) at 24 hpf. Water injections were performed in order to account for potential toxicity associated with injection-related stress. MO stock solutions (1 mM) were prepared by resuspending lyophilized MOs in molecular biology-grade (MBG) water, and stocks were stored at room temperature in the dark.

Working solutions of nc-MOs and pparγ-MOs were diluted to 0.5 mM in MBG water and working solutions of chd-MOs were diluted to 0.125 mM in MBG water. Newly fertilized (1- to 8-cell stage, or before 1.25 hpf) zebrafish embryos were microinjected with MOs (~three nL per embryo) using a motorized Eppendorf Injectman NI2 and FemtoJet 4x similar to previously described protocols (McGee et al., 2013; Dasgupta et al., 2017). At 3 hpf, MO delivery in embryos was confirmed using a Leica MZ10 F stereomicroscope equipped with a DMC2900 camera and a GFP filter cube; non-fluorescent and/or coagulated embryos were discarded. Fluorescent embryos were then exposed to either vehicle (0.2% DMSO) or 12.5 μM ciglitazone from 4 to 24 hpf and assessed for dorsoventral patterning abnormalities as described above.

To confirm pparγ knockdown, injected embryos (20 per pool; three replicate pools per group) were snap-frozen in liquid nitrogen at 24 hpf and stored at −80 °C. Total RNA was extracted using an SV Total RNA Isolation System (Promega, Madison, WI, USA) and eluted in 30 µL of nuclease-free water. RNA quality and quantity were confirmed using an Agilent 2100 Bioanalyzer system and Qubit 4.0 Fluorometer (Thermo Fisher Scientific, Waltman, MA, USA), respectively. A total of ~140 ng RNA per replicate sample was reverse-transcribed into cDNA using a GoScript Reverse Transcription System (Promega, Madison, WI, USA). An E1E2 pparγ fragment (~228 bp) was then amplified (forward primer: 5′-CACATCTACAGTAGTGCAGTCAT-3′; reverse primer: 5′-TGTTGGGTTGTTCTCGTAGTC-3′) using approximately 50 ng of cDNA per sample, ZymoTaq PreMix (Zymo Research, Irvine, CA, USA), and an Eppendorf Mastercycler Nexus Thermocycler with the following conditions: 2 min at 95 °C followed by 45 cycles of 95 °C for 30 s, 49.5 °C for 1 min, and 72 °C for 30 s. PCR products were visualized using an Agilent 2100 Bioanalyzer system.

### Oil Red O staining

To determine whether pparγ knockdown affected lipid homeostasis, embryos were injected with either water, nc-MOs, or pparγ-MOs, reared in particulate-free system water, and imaged under transmitted light at 6, 24, 48, 72, and 96 hpf. At each stage, a subset of embryos (seven per stage) were fixed in 4% paraformaldehyde (PFA)/1× phosphate-buffered saline (PBS) for 24 h and then transferred to 1× PBS. Fixed embryos were stained with ORO using previously described protocols (Passeri et al., 2009). Stained embryos were then imaged under transmitted light at 4× magnification using a Leica MZ10 F stereomicroscope equipped with a DMC2900 camera.

### Ciglitazone and DMP co-exposures

Embryos were exposed to either vehicle (0.2% DMSO), 12.5 μM ciglitazone, 0.078 μM DMP, or 12 μM ciglitazone + 0.078 μM DMP from 4 to 24 hpf in glass petri dishes (20 embryos per replicate per timepoint; three replicates per treatment). Maximum tolerated concentrations for DMP and ciglitazone co-exposures were optimized based on preliminary experiments that tested combinations of 0.078 or 1.56 μM DMP in the presence or absence of 9.375, 12.5, or 15 μM ciglitazone. Although 0.625 µM DMP was used in our prior studies (Dasgupta et al., 2017; Dasgupta et al., 2018), we relied on 0.078 µM DMP since co-exposure with 0.625 μM DMP and 12.5 μM ciglitazone resulted in a significant increase in mortality. At 8 hpf, embryos were fixed overnight in 4% PFA/1× PBS. Fixed embryos were manually dechorionated and then incubated overnight with anti-phosphoSMAD 1/5/9 IgG antibody (1:100 dilution; Cell Signaling Technology, Danvers, MA, USA) using previously described protocols (Yozzo, McGee & Volz, 2013; Dasgupta et al., 2018). Embryos were then incubated overnight with an IgG-specific Alexa Fluor-conjugated secondary antibody (1:500 dilution; Millipore Sigma, St. Louis, MO, USA). Embryos were imaged at 8× magnification using a Leica MZ10 F stereomicroscope equipped with a GFP filter and DMC2900 camera. At 24 hpf, embryos were imaged under transmitted light at 2× magnification using a Leica MZ10 stereomicroscope equipped with a DMC2900 camera and assessed for survival and dorsoventral patterning abnormalities as described above.

### mRNA-sequencing

To quantify potential effects of ciglitazone on the transcriptome, embryos were exposed to vehicle (0.2% DMSO), 12.5 μM ciglitazone, 0.078 μM DMP, or 12.5 μM ciglitazone + 0.078 μM DMP (20 embryos per dish; two dishes per replicate; four replicates per treatment) from 4 to 24 hpf and then immediately snap-frozen in liquid nitrogen at 24 hpf and stored at −80 °C. All embryos were homogenized in two mL cryovials using a PowerGen Homogenizer (Thermo Fisher Scientific, Waltman, MA, USA). Following homogenization, an SV Total RNA Isolation System (Promega, Madison, WI, USA) was used to extract total RNA from each replicate sample per the manufacturer’s instructions. RNA quantity and quality were confirmed using a Qubit 4.0 Fluorometer and 2100 Bioanalyzer system, respectively. Based on sample-specific Bioanalyzer traces, the RNA Integrity Number was >8 for all RNA samples used for library preparations.

Libraries were prepared using a QuantSeq 3′ mRNA-Seq Library Prep Kit FWD (Lexogen, Vienna, Austria) and indexed by treatment replicate per manufacturer’s instructions. Library quantity and quality were confirmed using a Qubit 4.0 Fluorometer and 2100 Bioanalyzer system, respectively. Raw Illumina (fastq.gz) sequencing files (16 files) are available via NCBI’s BioProject database under BioProject ID PRJNA544341, and a summary of sequencing run metrics are provided in Table S1 (>87.17% of reads were ≥Q30 across all runs). All 16 raw and indexed Illumina (fastq.gz) sequencing files were downloaded from Illumina’s BaseSpace and uploaded to Bluebee’s genomics analysis platform to align reads against zebrafish genome assembly GRCz10. After combining treatment replicate files, a DESeq2 application within Bluebee (Lexogen Quantseq DE1.2) was used to identify significant treatment-related effects on transcript abundance (relative to control) based on a false discovery rate *p*-adjusted value ≤ 0.05. Significantly affected transcripts were imported into the Database for Annotation, Visualization, and Integrated Discovery (DAVID) v6.8 for Gene Ontology (GO) enrichment analysis. Individual transcripts from significant GO terms (Benjamini score ≤ 0.05) were consolidated into a list of unique transcripts.

### Statistical analysis

For data derived from exposures and MO injections, a general linear model (GLM) analysis of variance (ANOVA) (α = 0.05) was performed using SPSS Statistics 24, as these data did not meet the equal variance assumption for non-GLM ANOVAs. Treatment groups were compared with vehicle controls using pair-wise Tukey based multiple comparisons of least square means to identify significant treatment-related differences.

## Results

### Zebrafish and mammalian PPARs are highly conserved

**Figure 1:**
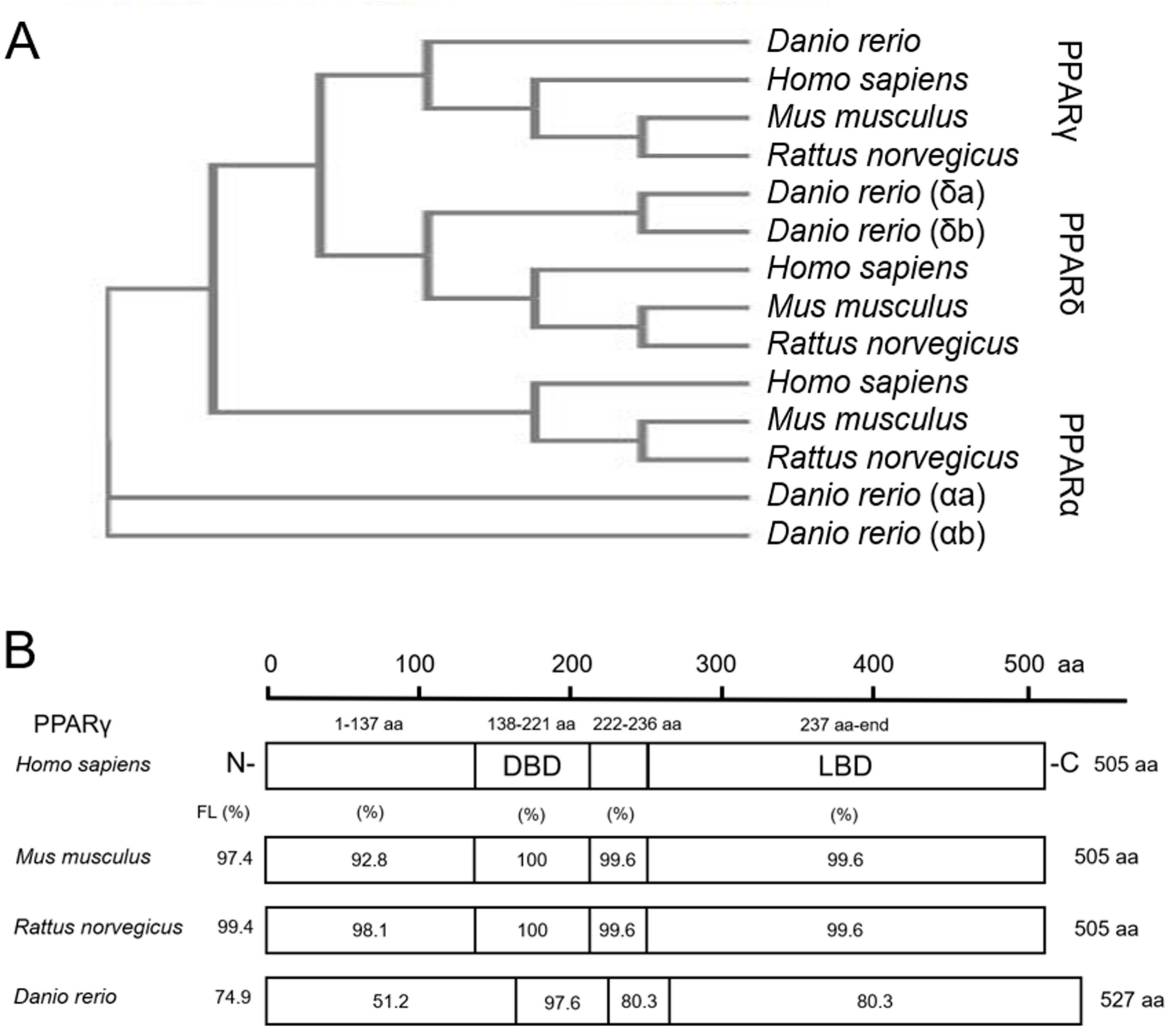
Zebrafish and mammalian PPARs are highly conserved. Phylogenetic tree showing relationship between *Homo sapiens* (human), *Mus musculus* (mouse), *Rattus norvegicus* (rat), and *Danio rerio* (zebrafish) PPARs (A). Percent similarity of mouse, rat, and zebrafish PPARγ relative to human PPARγ; FL, full length; DBD, DNA binding domain; and LBD, ligand binding domain (B).

As expected, when comparing protein sequences of PPARs from human, mouse, rat, and zebrafish, we found that zebrafish PPARα (a/b), PPARδ (a/b), and PPARγ are closely related to mammalian PPARα, PPARδ, and PPARγ, respectively (Fig. 1A). For each PPAR, the DNA binding domain (DBD) and ligand binding domain (LBD) were highly conserved across all four species (Fig. 1B; Fig. S1). For example, the percent similarity between full-length zebrafish and human PPARγ was 74.9%, whereas the similarity between the DBD and LBD was 97.6% and 80.3%, respectively (Fig. 1B)—a finding that was similar to PPARα and PPARδ (Fig. S1). When comparing similarity across zebrafish-specific PPARs, similarity in the LBD was highest (72.7%) between PPARγ and PPARδb (Fig. S2). Finally, when comparing ligand binding pocket amino acid residues involved with rosiglitazone binding to human PPARγ (Sheu et al., 2005), we found that eight out of 13 residues that interact with thiazolidinediones are conserved between humans and zebrafish (Fig. S3).

### Knockdown of pparγ adversely affects development within the first 96 h

**Figure 2:**
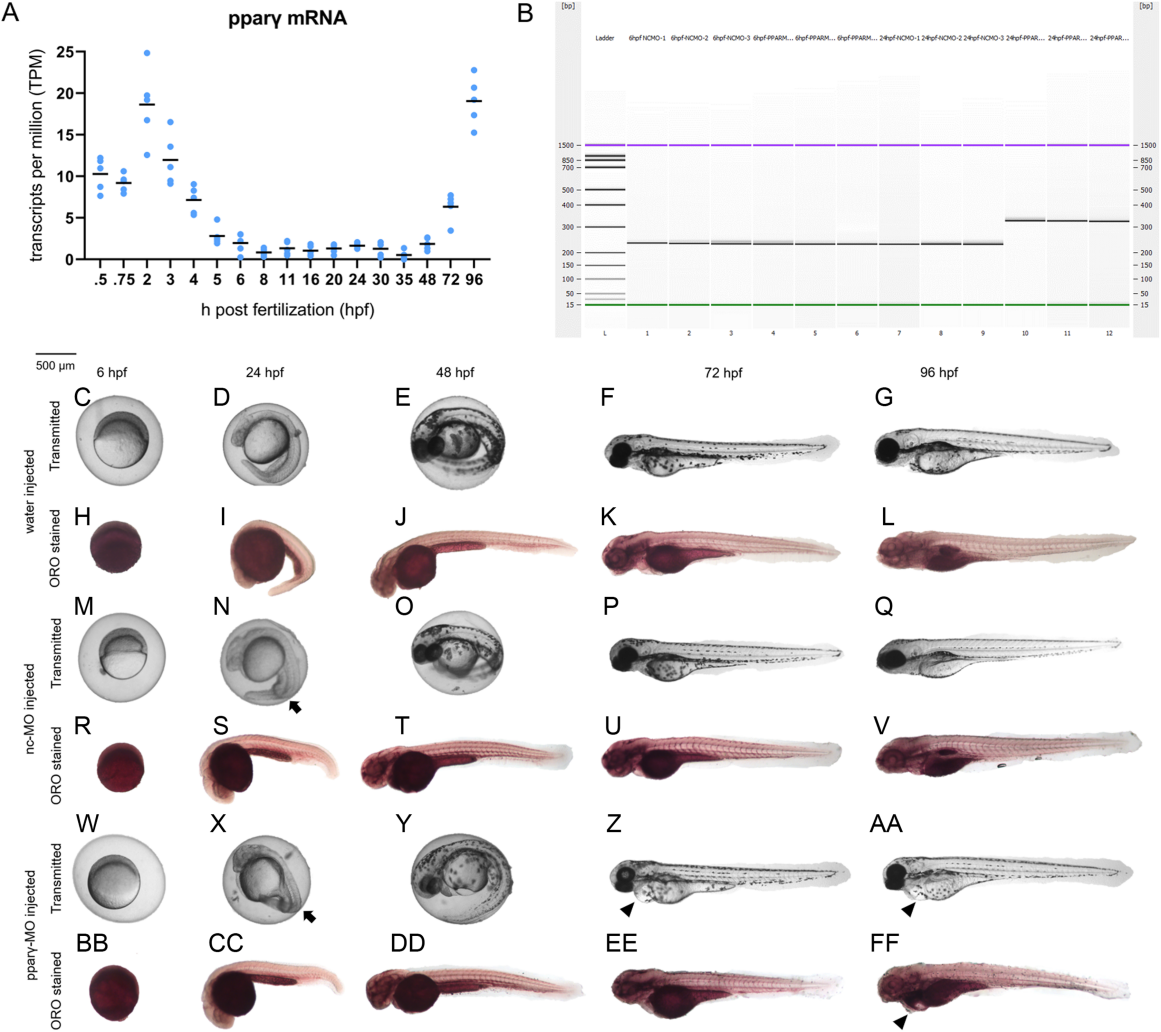
Knockdown of pparγ adversely affects development within the first 96 h. Abundance of pparγ mRNA within whole zebrafish embryos from 0.75 to 96 hpf (A). Confirmation of knockdown by 24 hpf following injection of splice-blocking pparγ-MOs. Lanes 1–3: 6-hpf embryos injected with nc-MO; lanes 4–6: 6-hpf embryos injected with pparγ-MO; lanes 7–9: 24-hpf embryos injected with nc-MO; and lanes 10–12: 24-hpf embryos injected with pparγ-MO (B). Representative images of water-, nc-MO-, or pparγ-MO-injected embryos from 6 to 96 hpf before and after staining with Oil Red O (ORO) (C–FF). Arrows point to mild dorsoventral patterning defects (ventralization), whereas arrowheads point to cardiac edema and cardiac looping defects.

Although there are elevated levels of pparγ transcripts between 0.75 and 5 hpf (due to maternally-loaded pparγ mRNA), zygotic transcription of pparγ mRNA does not occur until ~48 hpf (Fig. 2A). Similarly, maternally-loaded pparαa, pparαb, pparδa, and pparδb are all present within the embryo until 5 hpf (Fig. S4). While zygotic transcription of pparαa, pparαb, and pparδa is not initiated until ~24–30 hpf (Fig. S4), zygotic transcription of pparδb is initiated at 5 hpf and peaks at approximately 24 hpf (Fig. S4).

Injection of pparγ-MO resulted in insertion of intronic sequence within zygotic pparγ transcripts by 24 hpf (Fig. 2B). Within the first 48 h of development, injection of nc-MOs and pparγ-MOs resulted in mild defects on dorsoventral patterning relative to water-injected embryos (Figs. 2C–2FF). However, unlike nc-MO-injected embryos, injection of pparγ-MOs resulted in more severe developmental abnormalities including pericardial edema, cardiac looping defects, and stunted growth at 72 and 96 hpf (Fig. 2C–2FF)—a stage that coincides with a sharp increase in transcription of zygotic pparγ mRNA (Fig. 2A). However, the abundance of neutral lipids (as determined by ORO staining) within pparγ-MO-injected embryos was not qualitatively different across all stages (Fig. 2C–2FF).

### Knockdown of pparγ does not block ciglitazone-induced toxicity at 24 hpf

**Figure 3:**
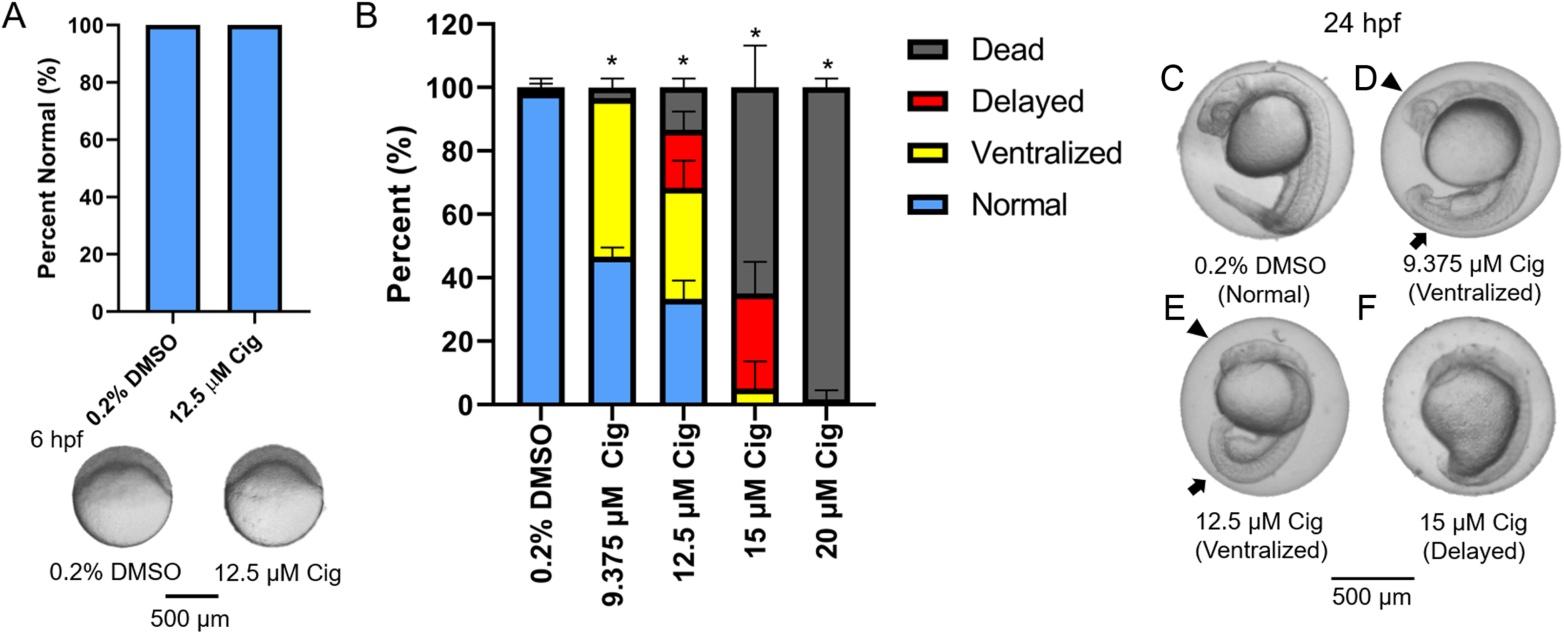
Initiation of ciglitazone exposure at 4 hpf results in dorsoventral patterning defects by 24 hpf. Initiation of ciglitazone (Cig) exposure at 4 hpf does not result in delayed epiboly by 6 hpf (*N* = 60 embryos per treatment) (A). Mean (± standard deviation) percent of normal, ventralized, dorsalized, or dead embryos following exposure to increasing concentrations of Cig from 4 to 24 hpf (*N* = 60 embryos per treatment) (B). Asterisk (*) denotes a significant difference (*p* < 0.05) in the percent of normal embryos relative to vehicle controls (0.2% DMSO). Representative images of (1) a normal embryo exposed to vehicle (0.2% DMSO) (C); (2) ventralized embryos exposed to 9.375 and 12.5 μM Cig (D and E); and (3) a delayed embryo exposed to 15 µM Cig (F). Arrows point to swollen yolk sac extensions, whereas arrowheads point to underdeveloped heads.

**Figure 4:**
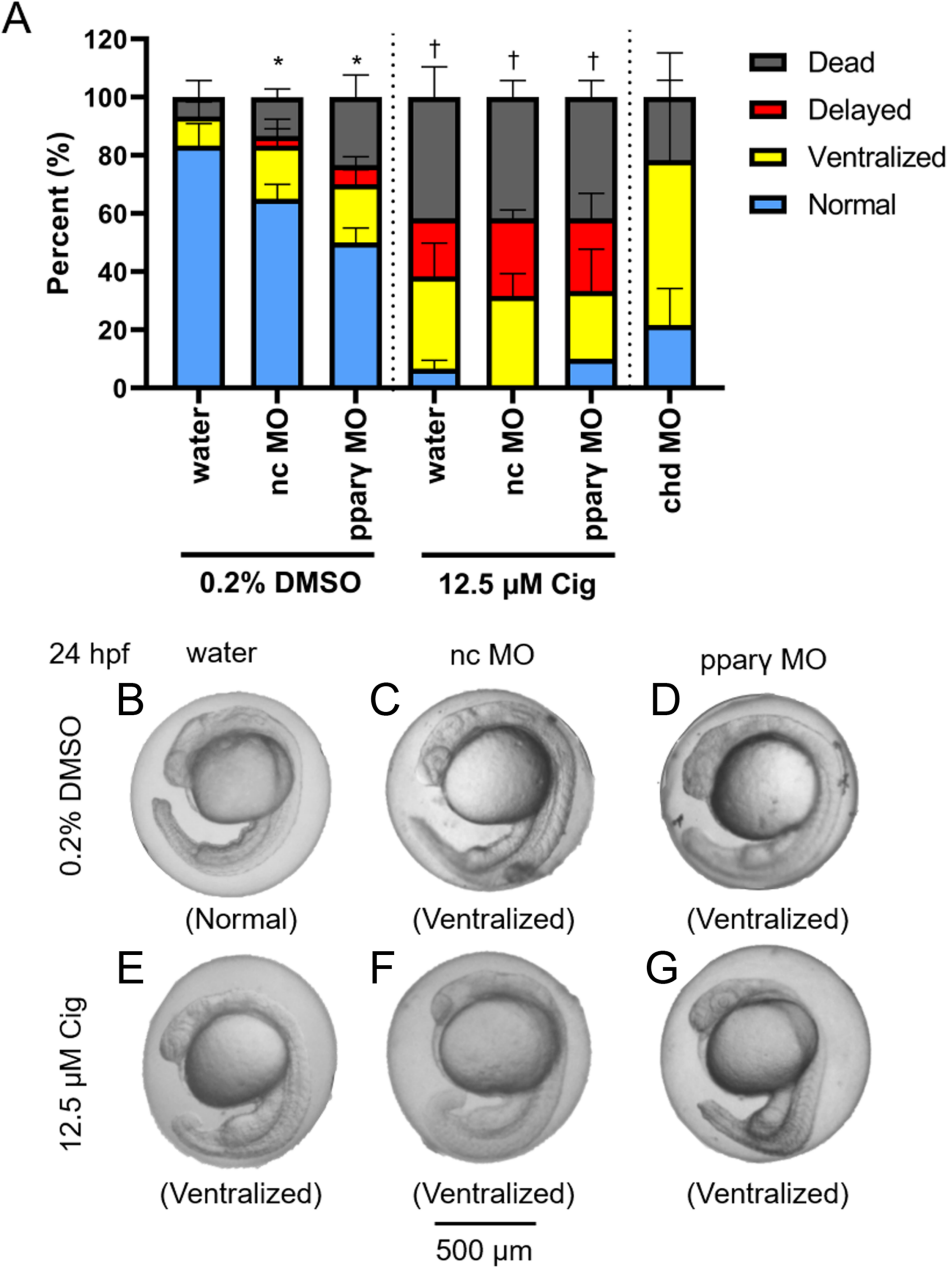
Knockdown of pparγ does not block ciglitazone-induced toxicity at 24 hpf. Mean (± standard deviation) percent of normal, ventralized, delayed, or dead embryos following injection of nc-MOs or pparγ-MOs at 0.75 hpf and exposure from 4 to 24 hpf to vehicle (0.2% DMSO) or 12.5 µM ciglitazone (Cig) (*N* = 60 embryos per treatment). Asterisk (*) denotes a significant difference (*p* < 0.05) in the percent of normal embryos relative to within-treatment water-injected controls (*p* < 0.05). Cross (†) denotes a significant difference (*p* < 0.05) in the percent of normal embryos relative to within-MO vehicle (0.2% DMSO) controls. chd-MO was used as a positive control for ventralization (A). Representative images of nc-MO- and pparγ-MO-injected embryos exposed to either vehicle (0.2% DMSO) or 12.5 µM Cig at 24 hpf (B–G).

Although ciglitazone exposure from 4 to 6 hpf did not impact epiboly at 6 hpf (Fig. 3A), initiation of ciglitazone exposure at 4 hpf resulted in a concentration-dependent effect on survival and dorsoventral patterning by 24 hpf (Figs. 3B–3F). Embryos injected with water, nc-MOs, or pparγ-MOs, and then exposed to vehicle (0.2% DMSO) from 4 to 24 hpf, exhibited mild effects on survival and dorsoventral patterning (Figs. 4A–4G). However, pparγ knockdown did not block dorsoventral patterning defects following exposure to ciglitazone from 4 to 24 hpf (Figs. 4A–4G).

### DMP reverses the ventralizing effects of ciglitazone

**Figure 5:**
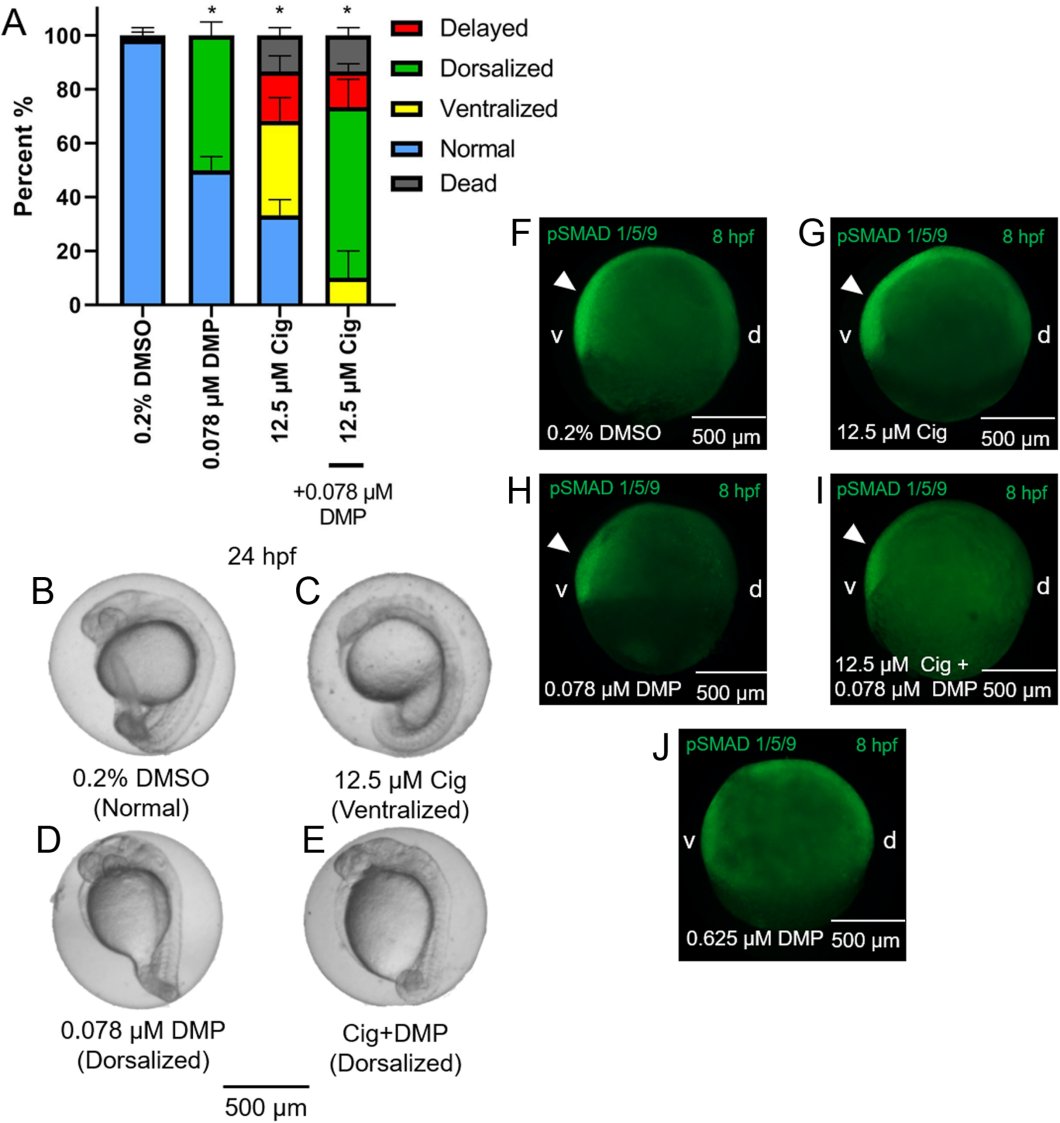
DMP reverses the ventralizing effects of ciglitazone. Mean (+standard deviation) percent of normal, ventralized, dorsalized, delayed, or dead embryos following exposure to vehicle (0.2% DMSO), 0.078 µM DMP, 12.5 µM ciglitazone (Cig), or 0.078 µM DMP + 12.5 µM Cig (*N* = 60 embryos per treatment) (A). Asterisk (*) denotes a significant difference (*p* < 0.05) in the percent of normal embryos relative to vehicle (0.2% DMSO) controls. Representative images of embryos following exposure to vehicle (0.2% DMSO), 0.078 µM DMP, 12.5 µM ciglitazone, or 0.078 µM DMP + 12.5 µM Cig (B–E). Immunostaining with anti-phosphoSMAD-1/5/9 within 8-hpf embryos following exposure to vehicle (0.2% DMSO), 0.078 µM DMP, 12.5 µM Cig, or 0.078 µM DMP + 12.5 µM Cig; embryos exposed to 0.625 µM DMP were included as a positive control for disruption of BMP signaling gradients. Arrowheads point to elevated pSMAD 1/5/9 staining on the ventral side of the embryo (F–J).

While exposure to 12.5 µM ciglitazone resulted in ventralized and delayed embryos, the majority of embryos following co-exposure with 12.5 µM ciglitazone + 0.078 µM DMP were dorsalized (Figs. 5A–5E). Although exposure to 0.625 µM DMP (a positive control) disrupted BMP signaling as expected (Fig. 5F–5J), exposure to 12.5 µM ciglitazone, 0.078 µM DMP, or 12.5 µM ciglitazone + 0.078 µM DMP did not result in disruption of BMP signaling at 8 hpf (Fig. 5F–5J).

### Ciglitazone exposure impacts cholesterol- and lipid-related biological processes by 24 hpf

**Figure 6:**
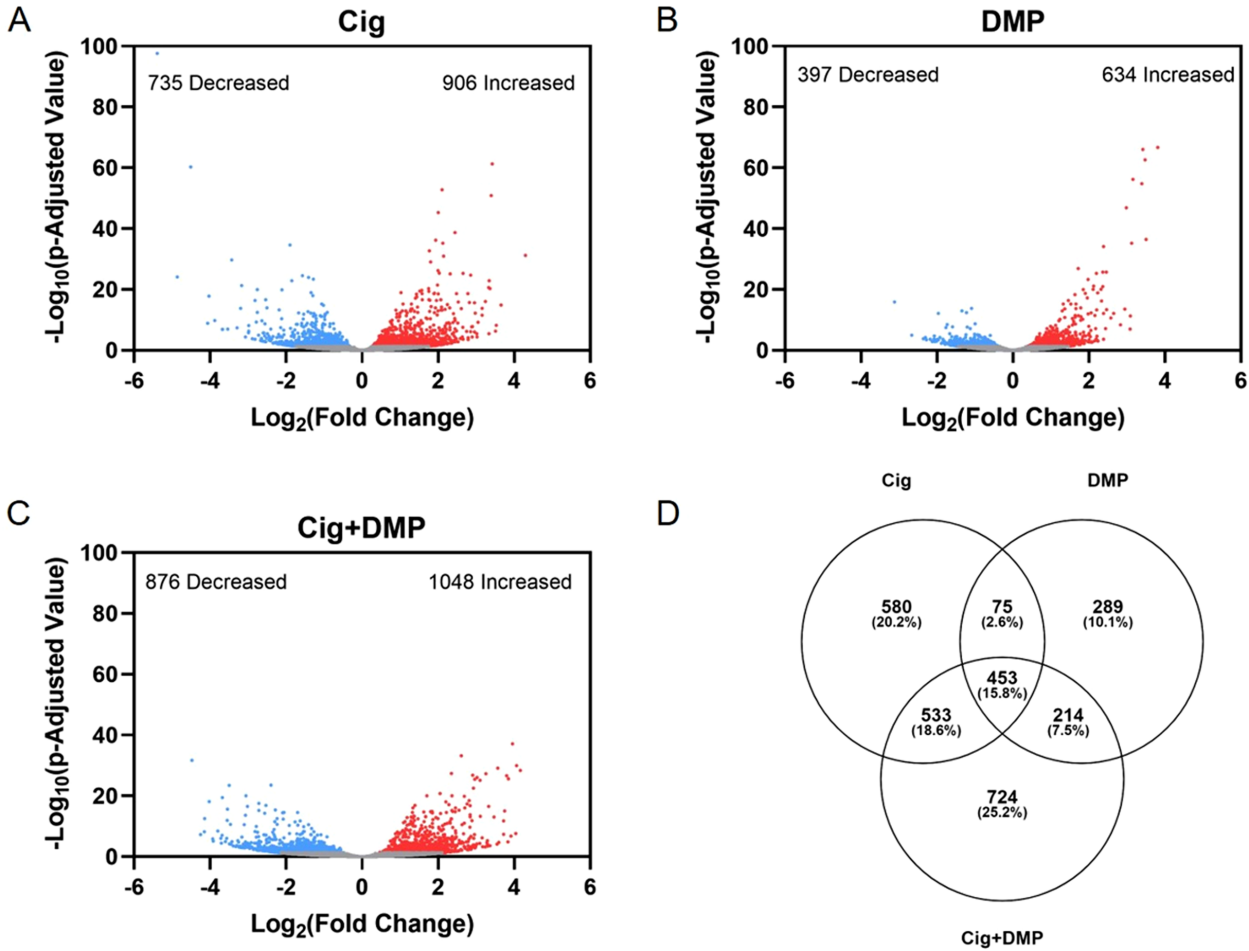
Exposure to ciglitazone, DMP, or ciglitazone + DMP results in significant effects on the transcriptome at 24 hpf. Volcano plots showing the number of significantly different transcripts following exposure to 12.5 µM ciglitazone (Cig) (A), 0.078 µM DMP (B), or 0.078 µM DMP + 12.5 µM Cig (C). All data are relative to vehicle (0.2% DMSO) controls. Log_2_ transformed fold change is plotted on the *x*-axis and log_10_ transformed *p*-adjusted value is plotted on the *y*-axis. Venn diagram showing the number and percent of significantly different overlapping transcripts among treatment groups; percentage values are relative to the total number of significantly different transcripts across all treatment groups (D).

**Figure 7:**
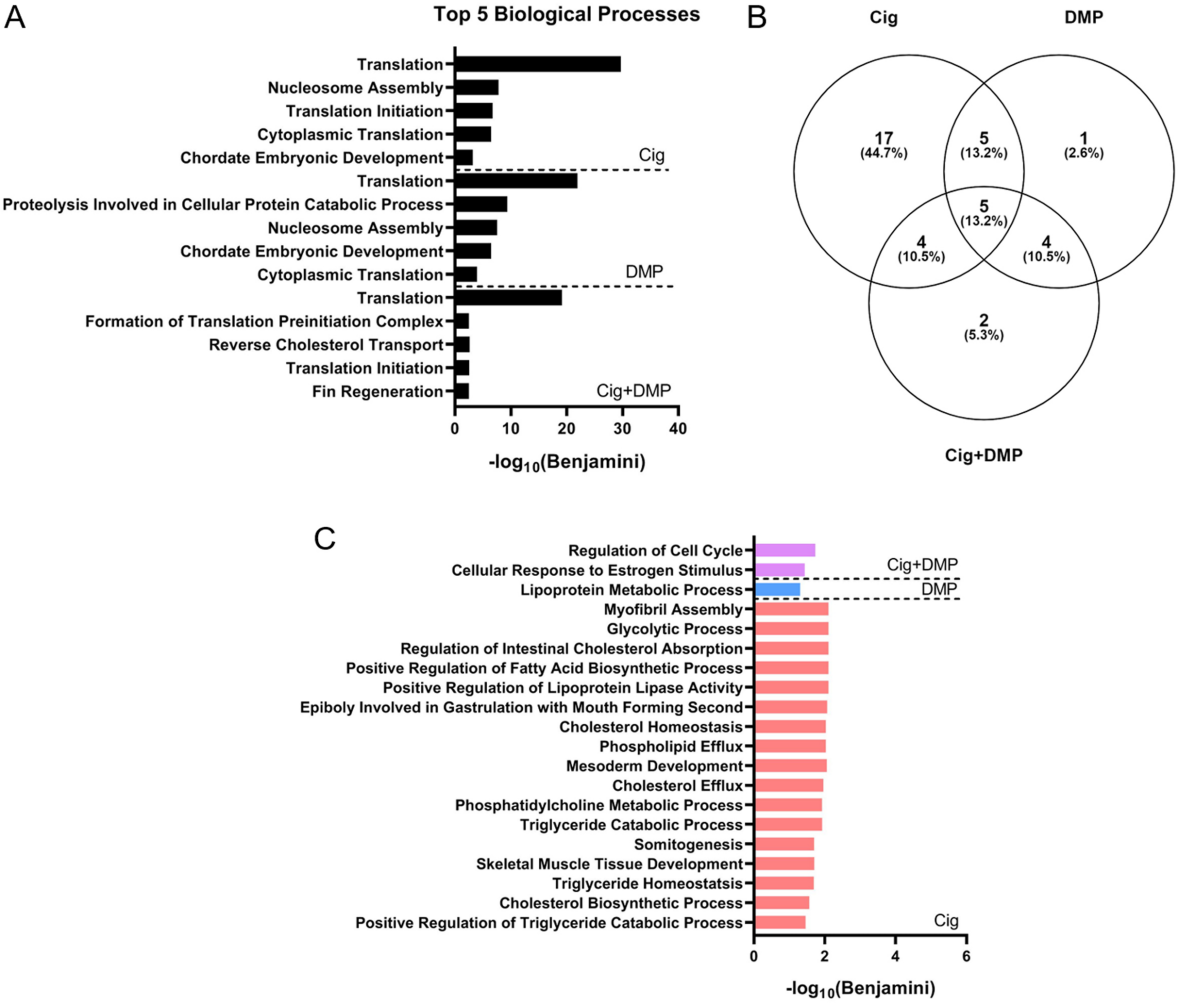
Ciglitazone exposure impacts cholesterol- and lipid-related biological processes by 24 hpf. Top 5 DAVID-identified biological processes (based on significantly different transcripts) following exposure to 12.5 µM ciglitazone, 0.078 µM DMP, or 0.078 µM DMP + 12.5 µM ciglitazone (A). Venn diagram showing the number and percent of significantly altered biological processes among treatment groups (B). Significant (Benjamini < 0.05) biological processes unique to each treatment group (C).

Exposure to 12.5 μM ciglitazone, 0.078 μM DMP, or 12.5 μM ciglitazone + 0.078 μM DMP resulted in a significant change in the abundance of 1,641, 1,031, and 1,924 transcripts, respectively, relative to vehicle controls (Figs. 6A–6C; Tables S2–S4). Although the magnitude of affected transcripts was similar across all three treatment groups, there was a total of 580, 289, and 724 significantly affected transcripts that were unique to embryos exposed to 12.5 μM ciglitazone, 0.078 μM DMP, or 12.5 μM ciglitazone + 0.078 μM DMP, respectively (Fig. 6D). Interestingly, the most significantly altered DAVID-based biological process across all treatment groups was translation (Fig. 7A; Tables S5–S7). While all three treatment groups shared certain biological processes that were affected (Fig. 7B; Fig. S5), exposure to ciglitazone alone primarily affected cholesterol- and lipid-related biological processes (Fig. 7C)—an effect that was driven by transcripts specific to apolipoprotein A-IV a (apoa4a), A-IV b (apoa4b.2), A-Ia (apoa1a), A-Ib (apoa1b), Eb (apoeb), antifreeze protein type IV (afp4), and an unnamed transcript (zgc: 162608) (Table S8).

## Discussion

Based on pairwise amino acid sequence alignments, zebrafish and mammalian PPARγ are strongly conserved, with the highest degree of homology located within the DBD and LBD. When comparing across PPARs, within-isoform homology was higher across species relative to within-species homology across isoforms. However, even with ~80% similarity in the LBD between human and zebrafish PPARγ, it is possible that deleted or altered amino acid residues in the thiazolidinedione binding pocket may affect the binding affinity or selectivity of ciglitazone to zebrafish PPARγ. Indeed, based on in vitro reporter assays comparing transactivation of human vs. zebrafish PPARγ, ciglitazone is unable to activate the LBD of zebrafish PPARγ within stably transfected reporter cell lines (HG5LN-GAL4-zfPPARγ)—a finding that is consistent with other thiazolidinediones (rosiglitazone, pioglitazone, and troziglitazone) (Riu et al., 2014). Despite similar functions in both humans and zebrafish, humans contain two functional isoforms of PPARγ (Li et al., 2016), a species-specific difference that may also account for potential differences in ciglitazone binding and activation of PPARγ within zebrafish.

Injecting embryos with nc-MO or pparγ-MO resulted in mild dorsoventral patterning defects compared to water-injected controls—a finding that may be due to off-target effects of both MOs. However, more severe effects from pparγ knockdown starting at 72 hpf—i.e., cardiac looping defects and pericardial edema—suggest that pparγ is necessary for later stages of embryonic development. Interestingly, these defects resulting from pparγ knockdown coincided with an increase in zygotic transcription of pparγ, suggesting that the delay in abnormalities within pparγ morphants was likely driven by zygotically-derived, unspliced pparγ pre-mRNAs. However, as we were unable to knockdown maternally-loaded pparγ transcripts (since we relied on a splice-blocking MO instead of a translational-blocking MO), it is unclear what role maternal pparγ mRNA may play within the first 5–6 h of development.

Ciglitazone was the first thiazolidinedione developed in the 1980s and was designed to be a potent and selective PPARγ agonist. Within in vivo studies, ciglitazone functions as an anti-hyperglycemic agent, inhibits human umbilical vein endothelial cell proliferation and angiogenesis, stimulates adipogenesis in preadipocytes, and decreases osteoblastogenesis in murine mesenchymal stem cells (Kawamatsu et al., 1980; Jozkowicz et al., 2002; Stephens et al., 1999; Lin et al., 2007). Based on dorosoventral patterning defects observed in this study, our findings suggest that, within developing embryos, ciglitazone may interact with other targets. Indeed, other thiazolidinedione compounds (i.e., pioglitazone and rosiglitazone) used to treat type two diabetes mellitus have also recently been withdrawn from the market due to off-target side effects such as fluid retention, increased risk of heart failure, and increased risk of bladder cancer (Nesto et al., 2003; Nissen & Wolski, 2007; Lewis et al., 2011). Therefore, it is possible that thiazolidinediones may have other mechanisms of action in addition to PPARγ activation.

Expression of BMP factors determine ventral cell fates within developing zebrafish embryos (Nikaido et al., 1997), whereas BMP antagonists (e.g., *noggin*, *follistatin*, and *chordin*) determine dorsal cell fates drive a gradient of BMP signaling (Smith & Harland, 1992; Hemmati-Brivanlou, Kelly & Melton, 1994; Sasai et al., 1994). In order to determine whether ciglitazone-induced dorsoventral patterning defects were mediated by BMP signaling, embryos were co-exposed to both ciglitazone and DMP (a BMP antagonist). We found that DMP reversed the ventralizing effects of ciglitazone, as embryos co-exposed to ciglitazone and DMP were primarily dorsalized. Moreover, in all treatment groups, pSMAD 1/5/9 localization was not disrupted relative to controls, indicating that ciglitazone-induced dorsoventral patterning defects were likely not due to a disruption in BMP signaling. This was further supported by our mRNA-seq data, as neither bmp2 nor psmad1/5/9 transcripts were significantly altered after exposure to 12.5 μM ciglitazone, 0.078 μM DMP, or 12.5 μM ciglitazone + 0.078 μM DMP. Similarly, exposure to tris(1,3-dichloro-2-propyl) phosphate (TDCIPP)—a high-production volume organohalogen flame retardant—induced dorsoventral patterning defects in the absence of effects on BMP signaling within developing zebrafish embryos (Dasgupta et al., 2018).

Our mRNA-seq data also demonstrated that exposure to ciglitazone resulted in significant alterations to lipid- and cholesterol-related biological processes. While it is unlikely that ciglitazone activates zebrafish PPARγ within the first 24 hpf (since pparγ knockdown did not rescue ciglitazone-induced defects at 24 hpf), pparδb may be a potential target of ciglitazone within developing zebrafish, as pparδb is ubiquitously expressed in all tissue types (Bertrand et al., 2007). Unlike other PPARs present in zebrafish, transcription of zygotic pparδb occurs during early stages of zebrafish development and zygotically-derived pparδb transcripts are elevated at 24 hpf. Within mouse and cell models, pparδb regulates fatty acid uptake, transport, and oxidation (Poirier et al., 2001; Holst et al., 2003)—biological processes that were significantly affected following exposure of zebrafish embryos to ciglitazone. In addition, based on pparδb-null mice, knockout of pparδb results in impaired growth and reduced gonadal adipose stores (Peters et al., 2000), showing that pparδb is necessary for proper development.

## Conclusions

Overall, we found that exposure of zebrafish embryos to ciglitazone resulted in dorsoventral patterning defects that are likely independent of PPARγ activation or disruption of BMP signaling. While PPARγ is required for normal embryonic development, the precise role of maternally-loaded PPARγ during early embryogenesis, as well as how zygotically-transcribed PPARγ may be mediating later developmental processes, remains unclear. Based on our findings to date, future studies should focus on determining whether (1) knockdown of maternally-loaded PPARγ impacts the first 24 h of development; (2) developmental abnormalities within zebrafish are driven by ciglitazone interaction with other targets or PPARs (such as PPARδb); and (3) ciglitazone exposure results in developmental abnormalities within mammalian models more relevant to human embryos.

## Supplemental Information

10.7717/peerj.8054/supp-1 Supplemental Information 1 Supplemental Figures S1–S5.

10.7717/peerj.8054/supp-2 Supplemental Information 2 Supplemental Tables S1–S8.

## Additional Information and Declarations

### Data Availability

The following information was supplied regarding data availability: The raw data is available as a Supplemental File.
