# Supplementary material for: Ciglitazone—a human PPARγ agonist—disrupts dorsoventral patterning in zebrafish
Source: PeerJ. 2019 Nov 13;7:e8054. doi: 10.7717/peerj.8054 (PMC6858815; doi:10.7717/peerj.8054)
Supplement: Supplemental Information 1 [file peerj-07-8054-s001.pdf]

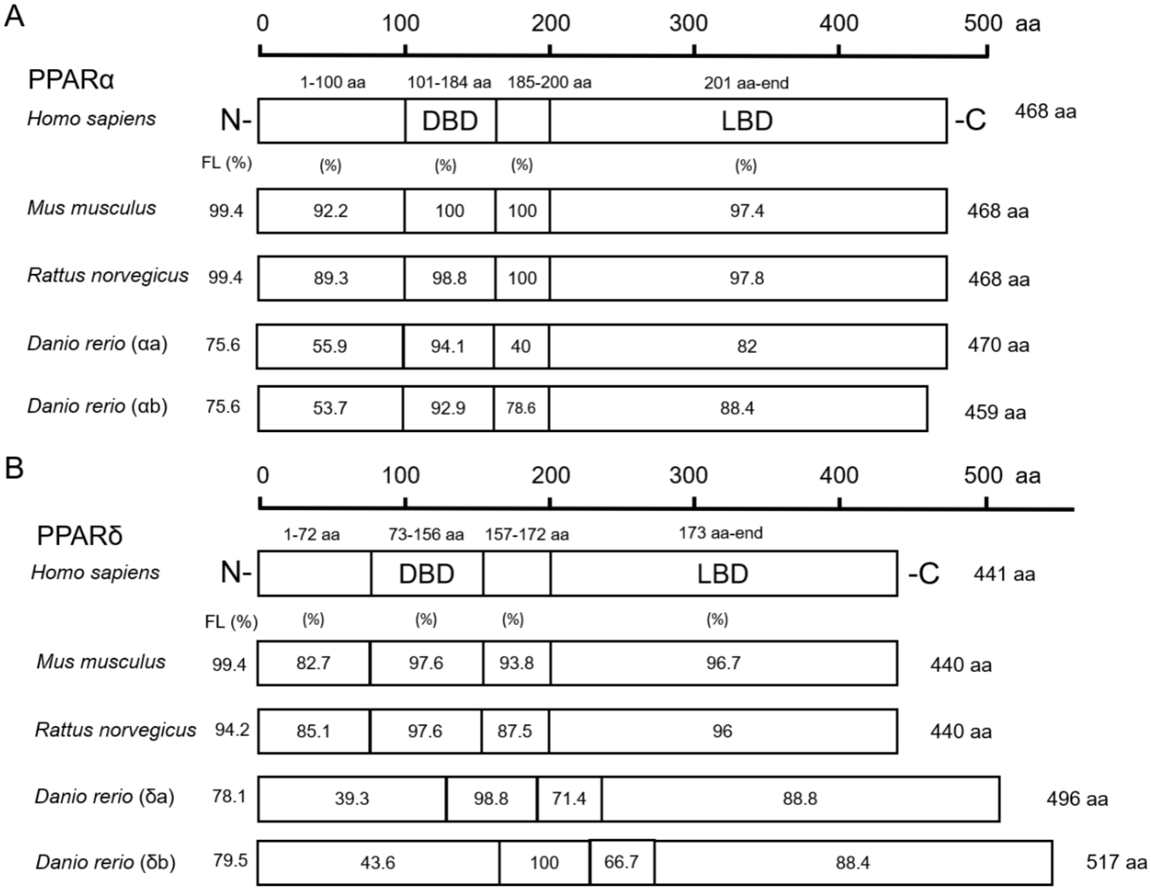

Figure S1. PPARα (A) and PPARδ (B) sequence similarity relative to human amino acid sequences. Percent similarity of mouse, rat, and zebrafish PPARα (A) and PPARδ (B) relative to human PPARα and PPARδ; FL = full length; DBD = DNA binding domain; and LBD = ligand binding domain.

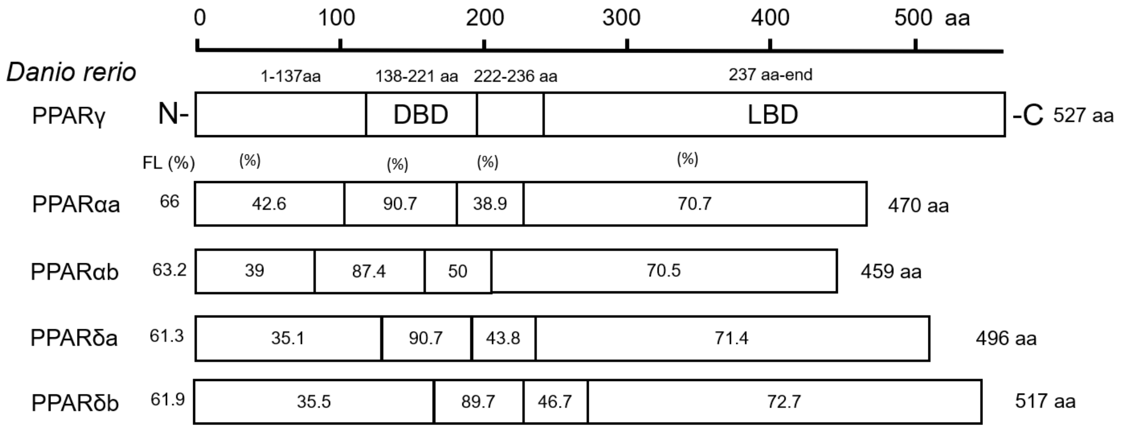

Figure S2. Percent similarity of zebrafish PPARα (a and b) and PPARδ (a and b) relative to PPARγ; FL = full length; DBD = DNA binding domain; and LBD = ligand binding domain (B).

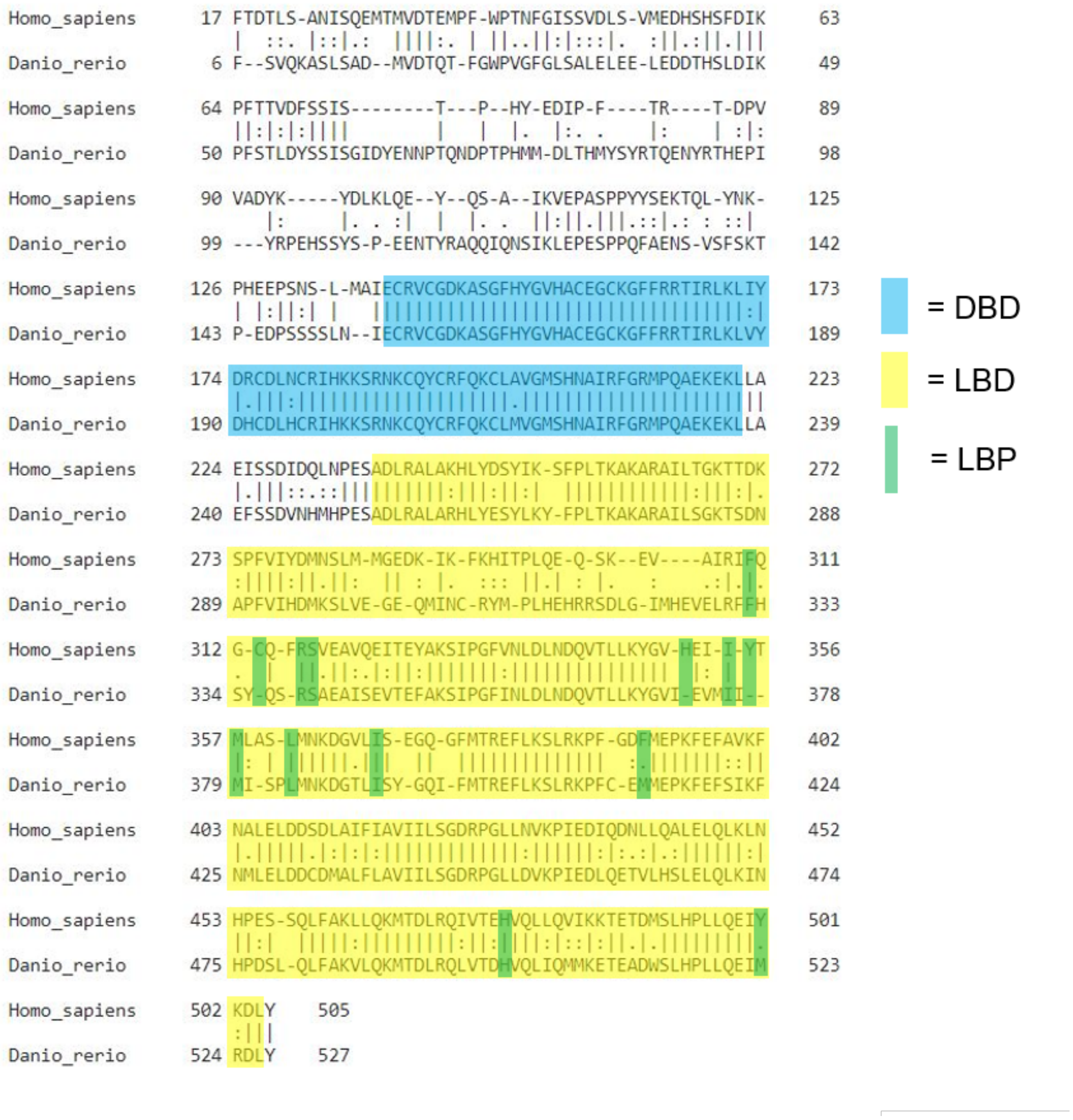

Figure S3. Sequence alignment between human and zebrafish PPARγ; DNA binding domain (DBD) is highlighted in blue, ligand binding domain (LBD) is highlighted in yellow, and amino acid residues in the ligand binding pocket (LBP) involved with thiazolidinediones binding are highlighted in green.

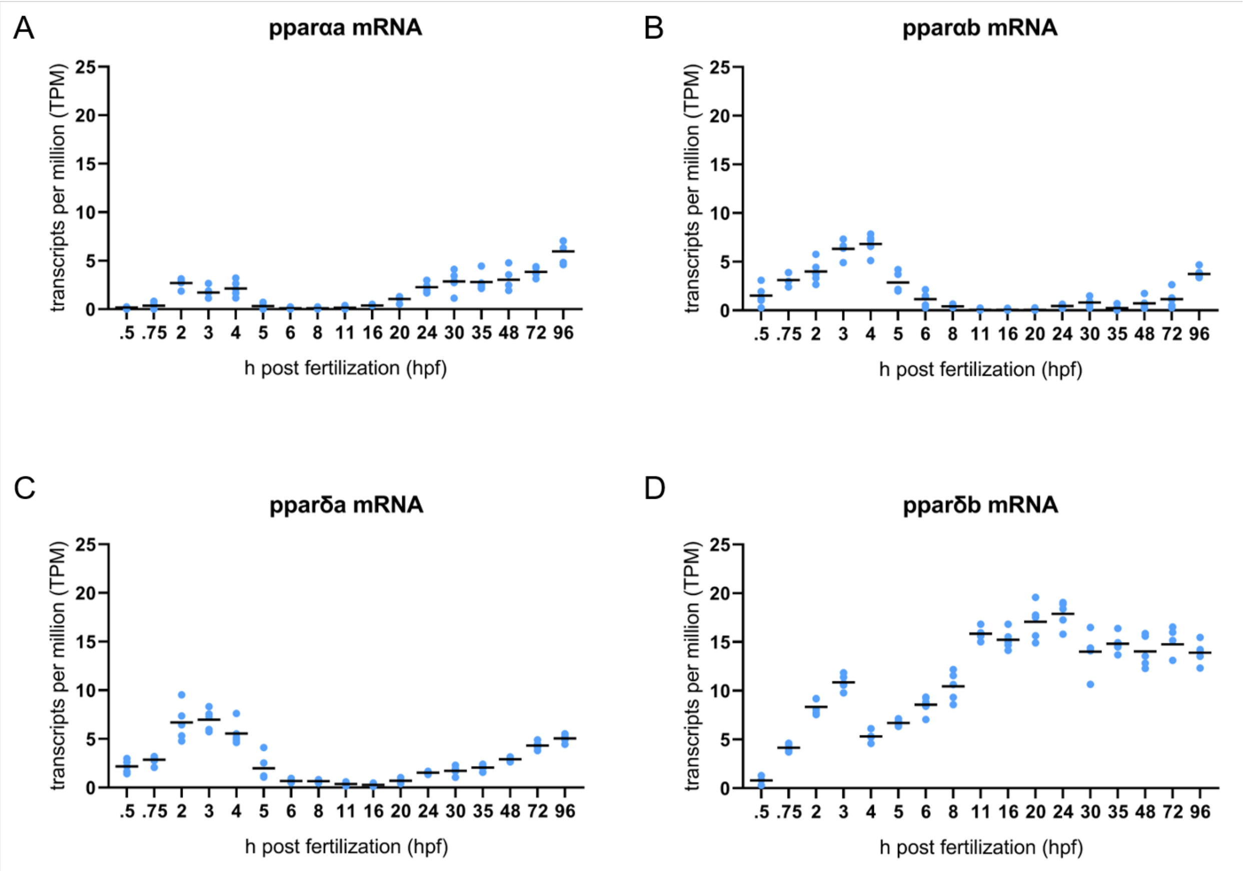

Figure S4. Abundance of pparαa (A), pparαb (B), pparδa (C), and pparδb (D) mRNA within whole zebrafish embryos from 0.75 hpf to 96 hpf.

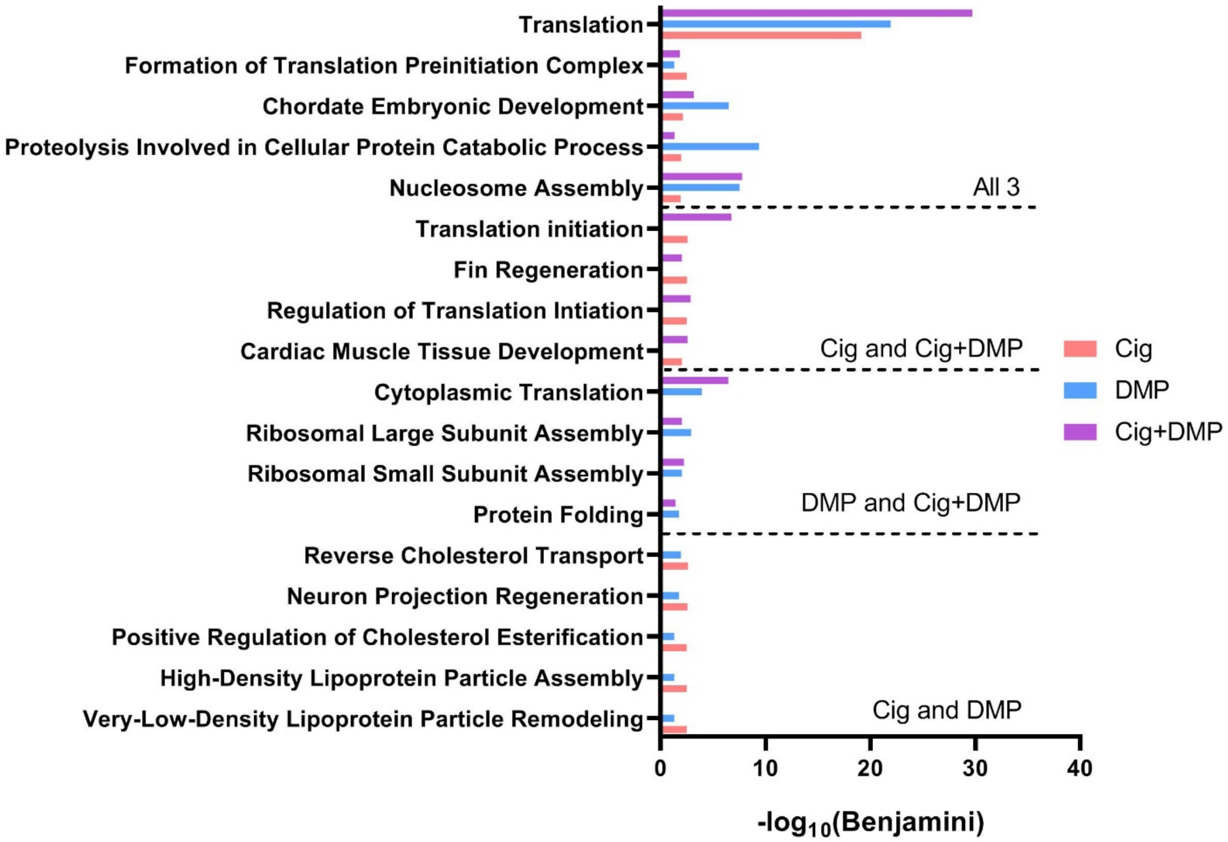

Figure S5. Significant (Benjamini p-value < 0.05) biological processes shared between or among treatment groups.
